# 668. Presence of the Narrow-Spectrum OXA-1 Beta-Lactamase Enzyme is not Associated with Elevated Ceftolozane-Tazobactam MIC Values among ESBL-Producing *Escherichia coli* Clinical Isolates (CANWARD, 2011-2018)

**DOI:** 10.1093/ofid/ofac492.720

**Published:** 2022-12-15

**Authors:** Andrew Walkty, James Karlowsky, Alyssa Golden, Philippe Lagace-Wiens, Melanie Baxter, Andrew Denisuik, Melissa McCracken, Michael Mulvey, Heather Adam, George Zhanel

**Affiliations:** University of Manitoba, Winnipeg, Manitoba, Canada; University of Manitoba, Winnipeg, Manitoba, Canada; University of Manitoba, Winnipeg, Manitoba, Canada; University of Manitoba, Winnipeg, Manitoba, Canada; University of Manitoba, Winnipeg, Manitoba, Canada; University of Manitoba, Winnipeg, Manitoba, Canada; National Microbiology Laboratory, Winnipeg, Manitoba, Canada; National Microbiology Laboratory, Winnipeg, Manitoba, Canada; University of Manitoba, Winnipeg, Manitoba, Canada; University of Manitoba, Winnipeg, Manitoba, Canada

## Abstract

**Background:**

Beta-lactam-beta-lactamase inhibitor combinations have been proposed as an alternative to carbapenems for the treatment of infections caused by extended-spectrum beta-lactamase (ESBL)-producing *Escherichia coli* as an antimicrobial stewardship initiative. However, in a recent randomized trial evaluating patients with bacteremia, presence of the narrow spectrum OXA-1 beta-lactamase enzyme among ESBL-producing Enterobacterales was associated with higher piperacillin-tazobactam MICs, and in turn excess mortality among patients treated with piperacillin-tazobactam relative to meropenem. The purpose of this study was to determine whether the in vitro activity of ceftolozane-tazobactam versus ESBL-producing *E. coli* is similarly compromised by the presence of OXA-1.

**Methods:**

*E. coli* clinical isolates were obtained from patients evaluated at hospitals across Canada (January 2011 to December 2018) as part of an ongoing national surveillance study (CANWARD). ESBL production was confirmed using the Clinical and Laboratory Standards Institute phenotypic method. Susceptibility testing was carried out using custom broth microdilution panels, and all isolates underwent whole genome sequencing for beta-lactamase gene detection.

**Results:**

In total, 485 ESBL-producing *E. coli* identified as part of the CANWARD study were included. The majority of isolates (91.3%; 443/485) harbored a CTX-M ESBL enzyme. OXA-1 was present in 39.6% (192/485) of isolates. OXA-1 was detected in 62.5% (187/299) of isolates with a CTX-M-15 ESBL enzyme versus only 2.7% (5/186) of isolates with other ESBL enzyme types. Ceftolozane-tazobactam MIC_50_ and MIC_90_ values were identical (0.25 µg/mL and 1 µg/mL, respectively) for the isolate subsets with and without OXA-1. Overall, 97.4% and 96.9% of isolates with and without OXA-1 remained susceptible (CLSI breakpoint) to ceftolozane-tazobactam.

**Conclusion:**

The presence of OXA-1 among ESBL-producing *E. coli* clinical isolates was not associated with an elevation in MIC values for ceftolozane-tazobactam. These data support further evaluation of ceftolozane-tazobactam as an alternative to carbapenems for the treatment of infections caused by ESBL-producing *E. coli*, as is being done with the MERINO-3 trial.

**Disclosures:**

**George Zhanel, PhD**, Merck: Grant/Research Support.

